# Organic acid-based deep eutectic solvents (DESs) are potent antimicrobial agents against antibiotic-resistant bacteria and viruses

**DOI:** 10.1016/j.crmicr.2026.100612

**Published:** 2026-05-26

**Authors:** Mohamed Abdelfattah Maky, Yanath Belguesmia, Chérifa Obone Opimba, Magloire Pandoua Nekoua, Toluwani Oshinowo, Mélissa Tourret, Rabah Boukherroub, Didier Hober, Djamel Drider

**Affiliations:** a UMR Transfrontalière BioEcoAgro INRAe 1158, Université de Lille, 59000 Lille, France; b Univ Lille, CHU Lille, Laboratoire de Virologie/ ULR3610, F-59000 Lille, France; c Univ. Lille, CNRS, Univ. Polytechnique Hauts-de-France, UMR 8520, IEMN, F-59000 Lille, France; d Department of Food Hygiene and Control, Faculty of Veterinary Medicine, Qena University, 83523 Qena, Egypt

**Keywords:** Deep eutectic solvents, Polymer nanoparticles, Antibacterial, Antivirals, Biofilms, Mechanism, Cytotoxicity

## Abstract

The escalating threat of antibiotic-resistant bacteria, biofilms and viral contamination in the healthcare and food industries threatens global health and requires the development of innovative antimicrobials. Deep eutectic solvents (DESs) have attracted attention as antimicrobial candidates, but there is little data on their effectiveness against antibiotic-resistant bacteria and viruses. This study evaluated the effectiveness of two organic acid-based DESs (T-glycoline and oxaline) and two non-organic acid-based DESs (reline and glyceline) against antibiotic-resistant Gram-positive and Gram-negative bacteria, as well as enveloped and non-enveloped viruses. Organic acid-based deep eutectic solvents outperformed their non-organic counterparts, achieving minimum inhibitory concentrations (MICs) of 0.20–0.83% v/v. T-glycoline and oxaline demonstrated synergy against colistin-resistant *Enterobacterales*, reducing the expression of the mobilised colistin resistance (*mcr*) gene. Combining T-glycoline with alginate/starch nanoparticles increased effectiveness against methicillin-resistant *Staphylococcus aureus* (MRSA). Organic DESs eradicated mature biofilms. Confocal microscopy revealed large numbers of dead bacterial cells, while transmission electron microscopy (TEM) showed cytoplasmic disruption. Combining alginate NPs with T-glycoline increased Caco-2 viability to 95% (compared to 15% with T-glycoline alone). T-glycoline and oxaline drastically reduced the infectious titres of both enveloped and non-enveloped viruses. Overall, these findings suggested that selected DES formulations may have potential as antimicrobial candidates, although their safety should be considered in practical applications.

## Highlights

• Organic acid-based DESs act against colistin- and methicillin-resistant strains.
• T-glycoline–nanoparticle mix enhances activity against MRSA.
• Organic DESs eliminate mature biofilms and damage bacterial cells.
• T-glycoline and oxaline reduce titres of enveloped and non-enveloped viruses.
• Nanoparticles enhance the safety of DESs.

## Graphical abstract

**Figure:**
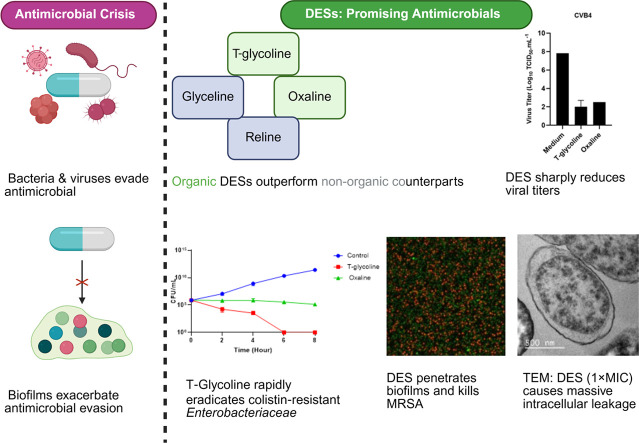

## 1 Introduction

The growing prevalence of drug-resistant bacteria and the formation of biofilms on medical devices and in hospital environments represents a serious problem, particularly in intensive care units where patients are highly susceptible to infection (Assefa and Amare, 2022). In these critical settings, antibiotic-resistant bacteria can proliferate and form biofilms that protect them from common antimicrobial treatments. This results in persistent infections that are difficult to eliminate. This problem also affects the veterinary sector, impacting feed storage, water supplies and milking machines. Poultry farms face serious challenges from biofilm development and bacterial resistance, particularly in environments where strict infection control is required (Piccirillo et al., 2024). Furthermore, drug-resistant bacteria thrive on food contact surfaces in catering and food preparation facilities, forming biofilms and becoming increasingly resistant to detergents. These biofilms compromise hygiene standards and increase the risk of contamination, particularly for ready-to-eat foods (Abebe, 2020).

Methicillin-resistant *Staphylococcus aureus* (MRSA) and colistin-resistant *Enterobacterales* are two of the most dangerous antibiotic-resistant, biofilm-forming bacteria. MRSA causes long-lasting and difficult-to-treat infections. It has several defence mechanisms, such as forming biofilms, which aid disease development and complicate treatment. MRSA associated with biofilms can cause a variety of infections, including osteomyelitis, bloodstream infections and endocarditis (Asgeirsson et al., 2018). MRSA is a significant concern in intensive care units, where many strains have become resistant to antibiotics. MRSA infections present a major obstacle to treatment, resulting in high morbidity and mortality rates across all age groups (Kaushik et al., 2024).

The *Enterobacterales* bacteria are serious foodborne pathogens responsible for a significant number of life-threatening infections in humans (Chlebicz and Śliżewska, 2018). *Enterobacterales* are a significant cause of hospital-acquired infections, often resulting in respiratory and urinary tract infections with high mortality rates (Peleg and Hooper, 2010). The formation of biofilms in *Enterobacterales* is a key virulence factor that enhances persistence and pathogenicity. These biofilms can develop in the human intestine, disrupting the gut microbiome and promoting the overgrowth of *Enterobacterales* (Lüthje and Brauner, 2014). Drug resistance in *Enterobacterales* is a global public health concern, caused by chromosomal gene mutations and the transfer of mobile resistance genes (Partridge, 2015). Colistin, a polypeptide antibiotic belonging to the polymyxin group, has been restricted for clinical use due to its toxicity. However, in recent years, it has been considered a last-line treatment for a range of infections caused by multidrug-resistant *Enterobacterales* (Ling et al., 2020). However, the therapeutic effectiveness of colistin has been compromised by the emergence of *mcr* genes (AbuOun et al., 2017). MCR genes can be transferred horizontally from one bacterium to another, resulting in a variety of bacterial species that are highly resistant to colistin (Ling et al., 2020).

Furthermore, numerous non-enveloped and enveloped viruses have been linked to nosocomial and healthcare-related infections through the faecal-oral, respiratory, and cutaneous-mucosal routes, as well as through exposure to blood, bodily fluids, infected tissues, and contaminated surfaces (Aitken and Jeffries, 2001). Their persistence on surfaces depends on temperature, humidity level, the presence of organic matter, and whether or not they have a viral envelope. They can persist for a few hours to several months (Alidjinou et al., 2017). Non-enveloped viruses generally survive better on surfaces than enveloped viruses (Firquet et al., 2015).

Therefore, there is an urgent need to develop and evaluate effective strategies against antibiotic-resistant bacteria, viruses, biofilms and persistent cells embedded within these protective communities. DESs have emerged as one of the most promising approaches to addressing these challenges. DESs are formed by mixing two or more components in a specific molar ratio to produce a eutectic mixture with a significantly lower melting point than that of the individual starting components (Durand et al., 2016). These mixtures can also form gels known as eutectogels (Meneses and Jesus, 2025). The physicochemical and biological characteristics of DESs depend on the nature of the parent compounds, the molar ratio, the temperature, and the percentage of water (Dai et al., 2015).

DESs have several desirable properties, including low flammability, simple preparation and low production expense. However, despite their clear potential, there is still debate regarding their toxicity. Recent critical review has emphasised that their toxicity to microbes is highly formulation‑dependent (Marchel et al., 2022). Furthermore, studies on DESs in air treatment suggest that some formulations containing toxic components can pose a hidden danger and may cause environmental pollution if not carefully managed (Janjhi et al., 2023). In addition, studies on hydrophobic DESS, including systems based on organic acids, indicate that traces of residual solvent and improper handling during water treatment can result in secondary pollution. This highlights the importance of carefully considering formulation stability and downstream disposal when applying these materials in practical applications (Marchel et al., 2023). This is particularly relevant for DESs based on organic acids, including systems containing oxalate. While these systems exhibit intriguing antimicrobial properties, their potential use in various applications must be considered alongside factors such as cytotoxicity, formulation stability and environmental impact.

One of the main areas of application for DESs is controlling harmful bacteria and viruses. Studies have demonstrated the effectiveness of DESs against various bacteria, including both Gram-positive and Gram-negative types (Nystedt et al., 2023; Bedair et al., 2024; Swebocki et al., 2024; Kocot et al., 2025) and against enveloped viruses, including HSV (Herpes simplex virus) (Siddiqui et al., 2023).

Although recent studies have recognised the antimicrobial potential of various DESs, existing literature has primarily focused on their broad-spectrum antibacterial activity or efficacy against specific pathogens (Swebocki et al., 2024). Despite these advances, a critical gap remains in their efficacy against antibiotic-resistant bacteria and the impact of these solvents on antibiotic resistance gene expression. Emerging studies have begun to explore their antiviral activity. However, current research largely focuses on enveloped viruses and the functional advantages of formulated organic acid-based DESs (Siddiqui et al., 2023). Consequently, the virucidal efficacy of organic acid-based DESs against non-enveloped human pathogens remains to be established.

This study addresses these gaps by evaluating the efficacy of DESs against antibiotic-resistant bacteria, their impact on resistance gene expression and their ability to eradicate biofilms and combat both enveloped and non-enveloped viruses. To elucidate the underlying mechanisms, we employ time-kill assays, transmission electron microscopy (TEM) imaging, and protein release quantification. Furthermore, we examine how the combination of DESs with nanoparticles (NPs) influences their antibacterial efficacy and their safety profiles.

## 2 Materials and methods

### 2.1 Chemicals and culture media

Two organic acid-based DESs, namely T-glycoline and oxaline, together with two non-organic acid-based DESs, reline and glyceline, were employed in this study. DESs were prepared following the previously described method (Table 1) (Swebocki et al., 2024). The chemicals used for their preparation included choline chloride (ChCl), urea (U), glycolic acid (GlycA), oxalic acid (OxA), glycerol (GLY), and tetrabutylammonium chloride (TBACl), all with high purity (≥99%) (Sigma-Aldrich, St. Louis, MO). All DES concentrations in this study are expressed as percentage by volume (% v/v).

**Table 1:** List of investigated DESs.

	DES	Parent substance 1	Parent substance 2	Density [g mL^−1^]	Reference
Organic acid	T-glycoline	TBACl	GlycA	1.14 ± 0.01	(Swebocki et al., 2024)
	Oxaline	ChCl	OxA	1.13 ± 0.01	(Abbott et al., 2004; Panhwar et al., 2018)
Non-organic acid	Reline	ChCl	U	1.17 ± 0.01	(Abbott et al., 2003)
	Glyceline	ChCl	GLY	1.18 ± 0.01	(Mjalli and Ahmed, 2017)

Here, TBACl: tetrabutylammonium chloride, GlycA: glycolic acid, ChCl: choline chloride, OxA: oxalic acid, U: urea and GLY: glycerol.

Colistin sulfate salt (Sigma-Aldrich, USA), Mueller–Hinton broth (BD Difco, USA), brain heart Infusion (BHI) medium (Sigma-Aldrich, St. Louis, MO, USA), tryptic Soy broth (Sigma-Aldrich, St. Louis, MO, USA) and cetyltrimethylammonium bromide (CTAB, SERVA, Germany) were used in this study.

### 2.2 Preparation of alginate and starch nanoparticles (NPs)

Alginate NPs were prepared by a top-down approach using a ball milling planetary mixer PM100 (RETSCH GmbH, Haan, Germany). Two grams of alginic acid sodium salt (Sigma Aldrich, St Louis, MA, USA) dried overnight at 70 °C using an oven (Memmert GmbH, Postfach), and 112 g of Zirconium oxide beads (3 × 20 mm + 10 × 10 mm) were placed in a 1.05 kg grinding jar in contact with each other for 10 h. Rotation speed was set at 440 rpm at room temperature, with a 10 min rotation interval and a subsequent 2 min pause.

Starch NPs were made from potato starch (Sigma Aldrich) with the same instrument but different protocol. 10 g of potato starch and 7 × 20 mm Zirconium oxide beads were placed in a 1.05 kg grinding jar for 2 h and was replaced with 16 × 10 mm Zirconium oxide beads for 3 h, where each hour represents a cycle. Rotation speed was set at 440 rpm at room temperature, with a 10 min rotation interval and a subsequent 2 min pause. Starch NPs were prepared and subjected to a grinding exposure time of five hours. All prepared NPs were stored in an air-tight container and kept in a cool and dry place.

### 2.3 Dynamic light scattering (DLS) measurements

The NPs were subjected to sonication using the ultrasonic bath (VWR Ultrasonic cleaner USC - THD), 3 min for starch and 10 min for alginate NPs. All batches of characterized NPs were diluted to 0.5 mg/mL in ultrapure water and analyzed in triplicate at 25 °C. Each experiment consisted of 12 runs per measurement with an applied voltage of 150 V, using a folded zeta capillary cell (DTS1070). Reproducibility of all measurements was verified by running triplicate measurements thrice. Every size reported herein is an average of nine results.

### 2.4 Bacterial strains

The bacterial strains used in this study are summarized in Table 2.

**Table 2:** Bacterial strains used in this study.

Strain	Antibiotic resistance gene	Source and/ or reference
*Escherichia coli* H45	Colistin (*mcr-1*) and β-lactam resistance	Clinical strain (Madi-Moussa et al., 2021)
*E. coli* H66	Colistin (*mcr-1*) and β-lactam resistance	Clinical strain (Madi-Moussa et al., 2021)
*E. coli* H184	Colistin (*mcr-1*)	Clinical strain (Madi-Moussa et al., 2021)
*Enterobacter cloacae* H51	Colistin (*mcr-9*) and β-lactam resistance	Clinical strain (Madi-Moussa et al., 2021)
*Salmonella enterica* H10	Colistin (*mcr-1*) and quinolone resistance	Clinical strain (Madi-Moussa et al., 2021)
*K. variicola* H77	Colistin (*mcr-9*) and β-lactam resistance	Clinical strain (Madi-Moussa et al., 2021)
*S. aureus* USA300	β-lactam and macrolides resistance	US(McDougal et al., 2003)
*S. aureus* S1	Methicillin resistance	Clinical strain (Belguesmia et al., 2021)
*L. monocytogenes* CIP 105,459		Wild-type reference strain
L. *monocytogenes* EGDe/pNZ-Phelp		Reference strain was used to elucidate pore forming mode of action (Crauwels et al., 2018)

### 2.5 Viruses and cells

Human epithelial cells HEp-2 (ATCC CCL-23) and Vero cells (African green monkey kidney cell line, ATCC CCL-81) were cultured at 37 °C under 5% CO₂ in MEM (Eagle's Minimal Essential Medium) supplemented with 1% penicillin, 1% non-essential amino acids, 1% l-glutamine, and 10% fetal calf serum.

CVB4, provided by Ji-Won Yoon (Julia McFarlane Diabetes Research Centre, Calgary, Alberta, Canada), was propagated in HEp-2 cells. HSV-1 (ATCC VR-260) was propagated in Vero cells. After 24 and 72 h of incubation of HEp-2 and Vero cells, respectively, the cell suspensions were frozen and thawed two times, followed by centrifugation (2000 × g for 10 min at 4 °C). The supernatants were then collected, aliquoted and stored at −80 °C. The viral titer in the culture supernatants was determined on HEp-2 and Vero cell cultures using the end-point dilution assay, and the Spearman-Kärber statistical method was used to calculate the 50% tissue culture infectious dose per mL (TCID₅₀/mL).

### 2.6 Minimum inhibitory concentrations (MICs)

The MICs were determined using the broth microdilution method according to the Clinical and Laboratory Standards Institute (CLSI) guidelines (M07-A13) and a previously reported protocol (Swebocki et al., 2023). Briefly, 100 µL of diluted DES in cation-adjusted Mueller-Hinton broth was added to each well of a microplate. Indicator strains were cultured in their suitable medium until reaching an optical density at OD_600_ of 0.1, then diluted 1:100 in Muller-Hinton broth. Subsequently, 50 µL of the diluted indicator strain was added to each well, and the plates were incubated overnight at 37 °C. MICs were defined as the lowest concentrations of DES that inhibited visible growth of the indicator bacteria.

MICs of colistin were determined using the broth microdilution method in cation-adjusted Mueller-Hinton broth, following the joint guidelines recommended by the European Committee on Antimicrobial Susceptibility Testing (EUCAST) and CLSI.

### 2.7 Minimum bactericidal concentration (MBC)

It was determined by transferring 10 μL aliquots from the clear wells onto fresh Muller-Hinton agar plates. These plates were then incubated at 37 °C for 48 h. After incubation, the plates were inspected for bacterial growth, and the MBC was defined as the lowest concentration at which no bacterial colonies appeared on the agar surface (Bajpai et al., 2016).

To determine whether the DES is bactericidal or bacteriostatic, the MBC/MIC ratio can be employed. If the MBC/MIC ratio is ≤4, the result is considered bactericidal. If the MBC/MIC ratio is greater than 4, the result is defined as bacteriostatic (Ishak et al., 2025).

### 2.8 Checkerboard assay for combining antimicrobials

Fractional inhibitory concentration index (FICI) was calculated for each combination using the subsequent equation: FIC = FIC_X_ + FIC_Y_ = (X/MIC_X_) + (Y/MIC_Y_), where (X) is the lowest level of antimicrobial X in combination with another to produce an inhibitory impact, while (MIC_x_) is the MIC of that antimicrobial alone for the bacterial strain under study. FIC index findings are categorized as following: FIC ≤ 0.5 is synergy, 0.5 < FIC ≤ 0.75 is partial synergy, 0.75 < FIC ≤ 1.0 is additive, FIC >1.0 is indifferent and FIC > 4 is antagonistic (Petersen et al., 2006).

### 2.9 Evaluation of antibacterial activity of DES-loaded alginate and starch nanoparticles (NPs)

To investigate the impact of NPs incorporation on the antibacterial activity of DESs, suspensions of alginate and starch NPs were prepared. Each suspension was sonicated at 25 °C for 60 min to achieve a uniform concentration of 500 µg/mL. These suspensions were then combined with T-glycoline and oxaline at a final concentration of 20% (v/v), achieving a total volume of 300 μL through vortex mixing to ensure homogeneity.

### 2.10 The impact of different DES formulations on the expression of colistin resistance genes

It was assessed using RT-qPCR. *E. coli* H45 (carrying *mcr*-1) and *Enterobacter cloacae* H51 (carrying *mcr*-9) were treated with colistin and DESs at sub-inhibitory concentrations (half the MIC) for 24 h. Total RNA was then extracted from the treated cells using a NucleoSpin™ RNA kit (Macherey-Nagel, Düren, Germany), following the manufacturer's protocol. One microgram of RNA from each sample was treated with DNase (Thermo Fisher Scientific) and then transcribed into complementary DNA (cDNA) using the RevertAid H Minus First Strand cDNA Synthesis Kit (Thermo Fisher Scientific), following the manufacturer's instructions. Quantitative PCR reactions were prepared with a total volume of 20 µL, consisting of 10 µL Takyon™ No ROX SYBR 2X MasterMix blue dTTP (Eurogentec, Seraing, Belgium), 1 µL of gene-specific primers (Table 3), 2.5 µL of cDNA template, and 5.5 µL of nuclease-free water (Madi-Moussa et al., 2021). Amplifications were performed on a CFX Connect Real-Time PCR Detection System (Bio-Rad, Hercules, CA, USA). The thermal cycling protocol started with an initial denaturation at 95 °C for 10 min, followed by 45 cycles of 95 °C for 15 s for denaturation, 56 °C for 15 s for primer annealing, and 72 °C for 20 s for extension. A melting curve analysis ranging from 90 to 58 °C was subsequently conducted to confirm the specificity of the amplified products (Postollec et al., 2011). Cycle threshold (C_t_) values were determined using Bio-Rad’s CFX Manager software. Relative gene expression of the target *mcr* genes was calculated against the 16S rRNA housekeeping gene using the 2^−(∆∆Ct)^ method, as previously described (Livak and Schmittgen, 2001).

**Table 3:** Oligonucleotide primers used for RT-qPCR analysis.

Name	Sequences	Target gene	Size (bp)
16S rRNA-F	TCGGAAACGGACGCTAATAC	16S rDNA	191
16S rRNA-R	CGTAGGAGTCTGGACCGTGT		
*mcr*-1-F	CGCGATGCTACTGATCACCA	*mcr*-1	100
*mcr*-1-R	AAAATAACTGGTCACCGCGC		
*mcr*-9-F	ATCCGTTCCGTGCATGTTCT	*mcr*-9	100
*mcr*-9-R	CACCGGTTTTCTGCACGATG		

### 2.11 Antibiofilm assay

We evaluated the efficacy of DESs in eradicating biofilms formed by the Gram-positive bacterium *S. aureus* S1 and the Gram-negative bacterium *E. coli* H45, according to the manufacturer's instructions using a commercial biofilm formation assay kit (Dojindo Laboratories, Fukuoka, Japan). *S. aureus* S1 cultures were grown in tryptic soy broth supplemented with 1% glucose, while *E. coli* H45 cultures were grown in brain heart infusion medium to an OD_600_ of 0.1. A sample of 180 µL of each bacterial suspension was transferred to individual wells of a 96-well microtiter plate. The plate was covered with a polystyrene pin lid (Nunc-Immuno TSP; Thermo Fisher Scientific, Massachusetts, USA) and incubated at 37 °C for 48 h to allow biofilm formation. After incubation, the pin lids were washed with sterile physiological saline and transferred to a fresh 96-well plate containing medium supplemented with DESs at twice the MIC. The plates were then incubated at 37 °C for three hours. Following treatment, the pin lids were washed again and placed in wells containing 200 µL of 0.1% crystal violet solution for 30 min to stain the biofilms. Excess dye was removed by additional washing. The stained biofilms were then extracted into 200 µL of ethanol per well for 15 min. Biofilm biomass was quantified by measuring the absorbance of the extracted crystal violet at 600 nm using a microplate reader. Biofilm eradication efficiency was calculated using the following equation (Long et al., 2022):

Biofilmeradication(%)=[1−(ODoftestDES/ODofcontrol)]×100%.

### 2.12 Live/dead confocal microscopy

Biofilms of *S. aureus* S1 and *E. coli* H45 were established by culturing cells in Labtek chambers (Dominique Dutscher SAS, Bernolsheim, France) in tryptic soy broth supplemented with 1% glucose and BHI medium, respectively, at 37 °C for 48 h. The mature biofilms were then treated with DESs at twice the MIC, followed by incubation at 37 °C for three hours. After treatment, the biofilms were gently washed with sterile physiological saline to remove planktonic cells and residual medium, and stained with Syto-9 and propidium iodide, in accordance with the protocol for the FilmTracer™ LIVE/DEAD® Biofilm Viability Kit (Invitrogen, Carlsbad, CA, USA), without concentrating the cells prior to imaging. The stained samples were then visualised using a Zeiss LSM 700/780 confocal laser scanning microscope equipped with ZEN 2010 software (Oberkochen, Germany).

### 2.13 Time-kill kinetics assay

It was performed to evaluate the survival kinetics of selected Gram-positive and Gram-negative bacterial strains in the presence of the DES formulations. The Gram-positive strains included *S. aureus* USA300 and *S. aureus* S1, while the Gram-negative strains comprised *E. coli* H45 and *Enterobacter cloacae* H51. Each strain was cultured in its appropriate growth medium until reaching an optical density at OD_600_ of 0.1, followed by a 100-fold dilution. The cultures were then incubated with the DES formulations at a concentration equivalent to 1 × MIC. At predetermined time intervals, samples were collected, serially diluted, and plated onto brain heart infusion (BHI) agar. Colony-forming units per milliliter (CFU/mL) were determined after incubation for 24 h at 37 °C (Belguesmia et al., 2020).

### 2.14 Transmission electron microscopy (TEM)

Cultures of *S. aureus* USA300, and *S. aureus* S1, *E. coli* H45 and *Enterobacter cloacae* H51 were grown in Muller-Hinton broth until reaching an OD_600_ of 0.1. The bacterial cultures were then treated with the DES formulations at a concentration equivalent to 1 × MIC and incubated overnight at 37 °C. Cells were harvested by centrifugation at 10,000 × g for 10 min at 5 °C, washed with phosphate-buffered saline (PBS), and fixed with 2.5% (v/v) glutaraldehyde in 0.1 M cacodylate buffer (pH 7.4). Fixed cell pellets were placed onto Formvar-coated 300-mesh nickel grids (EMS, FF300-Ni). TEM images were acquired using a JEOL JEM-2100FX transmission electron microscope operated at an acceleration voltage of 200 kV, equipped with GATAN CCD Orius 1000 and GATAN CCD Orius 200D cameras.

### 2.15 Quantification of extracellular protein leakage following DESs treatment

The intracellular protein content of *S. aureus* USA300*,* and *S. aureus S1, E. coli H45 and Enterobacter cloacae* H51 was quantified using the QuantiPro™ BCA Assay Kit (Sigma-Aldrich), following treatment of the bacterial cells with DESs. In brief, two millilitres of overnight cultures of *S. aureus* USA300*, and S. aureus* S1*, E. coli* H45 *and Enterobacter cloacae* H51, grown in Muller Hinton broth, were centrifuged at 9000 g for 10 min at 4 °C. The supernatant was discarded, and the resulting cell pellets were washed and resuspended in sterile phosphate-buffered saline (PBS, pH 7.0). After a second centrifugation under the same conditions, the pellets were treated with 2 mL of the respective DESs solutions at a concentration equivalent to 1 × MIC. The treated samples were incubated for 2, 4, 6, 8 and 24 h. At the end of each incubation period, the samples were centrifuged again to collect the supernatant (Belguesmia et al., 2020). A standard protein curve was prepared using bovine serum albumin (BSA) at various concentrations according to the manufacturer’s instructions. The protein concentration in each supernatant was determined by mixing the samples with QuantiPro reagents and incubating them for one hour at 60 °C. After incubation, the absorbance was measured at 578 nm using a SAFAS MP96 Microplate Reader (SAFAS S.A., Monaco). Extracellular protein concentrations were then calculated based on the standard BSA calibration curve.

### 2.16 Investigation of the pore-forming activity of DESs

This was assessed using fluorescence measurements with the *L. monocytogenes* EGD-e/pNZ-Phelp-pHluorin. *L. monocytogenes* EGD-e/pNZ-Phelp-pHluorin cultures were grown overnight in BHI medium, washed with PBS, and then resuspended in a filter-sterilised LMB with a 0.2 μm pore size. Aliquots of the bacterial suspension (100 μL) were mixed with 100 μL of LMB containing DESs at 1 × and 2 × MICs in microtiter plate wells. Untreated bacteria cultured in LMB were used as the negative control, while 0.01% (v/v) of CTAB was used as the positive cytotoxic control. After an hour incubation at room temperature, fluorescence emission was recorded at 510 nm following excitation by scanning wavelengths from 350 to 490 nm, or at the pHluorin excitation maxima of 400 and 470 nm, using a SpectraMax microplate reader (Thermo Fisher Scientific, Waltham, MA, USA) (Crauwels et al., 2018).

### 2.17 Virucidal activity of DESs

Cytotoxicity assays were first performed using HEp-2 and Vero cells (2.5 × 10⁴ cells/well) cultured in 96-well plates at 37 °C under 5% CO₂ for 24 h. The culture medium was then removed and different concentrations (% v/v) of T-glycoline, glyceline, reline or oxaline diluted in MEM were added to the cells. The HEp-2 and Vero cells were then incubated again for 24 h, and cell viability expressed as a percentage compared to untreated cells was assessed using the Orangu™ assay (Cell Guidance Systems) according to the manufacturer's instructions. The CC_50_ was determined with GraphPad Prism software (version 8.0.2) using a nonlinear regression analysis.

The virucidal activity of DESs was evaluated against HSV-1 and CVB4. Each DES at 10% (v/v) was incubated with HSV-1 or CVB4 for one hour, and the mixtures were then diluted 1:10 to overcome the cytotoxic concentration. The infectious titer in the different dilutions with DES at 1% was then determined on HEp-2 or Vero cell cultures using the end-point dilution assay. Briefly, a 10-fold dilution series was carried out in culture medium to reach DES concentrations from 10^−1^ to 10^−8^%. Then, 100 µL of each dilution were distributed in six replicates in 96-well plates containing 1.5 × 10⁴ HEp-2 or Vero cells. The plates were incubated for 48 to 96 h at 37 °C and 5% CO_2_. The cytopathic effect (CPE) was then read using an Olympus CKX41 microscope and the Spearman-Kärber statistical method was used to calculate the 50% tissue culture infectious dose per mL (TCID₅₀/mL).

### 2.18 Safety assessment

Both cytotoxicity and haemolytic activity were carried out to assess the safety profile of DESs and their formulations with alginate and starch nanoparticles.

*Cytotoxicity assay:* The effects of DES dissolved in fresh DMEM, either alone or adsorbed on NPs, were evaluated using a Caco-2 cell culture model. Cells were cultured at 37 °C in a humidified atmosphere containing 5% CO₂ in DMEM with 4.5 g. l^-^¹ glucose (PAN-Biotech GmbH, Aidenbach, Germany), supplemented with 10% fetal bovine serum, 100 U.mL^-^¹ penicillin, 100 μg.mL^-^¹ streptomycin, and 2 mM glutamine. Cells were seeded in 96-well tissue culture plates at a density of 8000 cells per well and allowed to grow for five days. For cytotoxicity assessment, 100 μL of each sample at the appropriate concentration was added to the wells, and the cells were incubated for an additional 24 h. Cell viability was then determined using the CCK-8 assay (Tebubio, Le Perray-en-Yvelines, France) by adding 25 μL of CCK-8 reagent (final concentration 5%) to each well containing 100 μL of sample. After incubation in the dark for 90 min, absorbance was measured at 450 nm using a SpectraMax® iD3 spectrophotometer (Molecular Devices, San José, CA, USA). Results were expressed as a percentage of the viability of untreated control cells.

*Hemolytic activity*: It was assessed on TSA with 5% sheep blood (Thermo Scientific™, Germany). Aliquots (10 µL) of T-glycoline and oxaline (each at 0.5% v/v), either alone (sterile Milli-Q water) or combined with alginate or starch NPs, were spotted onto the plates. Plates were incubated aerobically at 37 °C for 24 h, with CTAB (0.02% v/v) as the positive control and sterile Milli-Q water as the negative control (Maragkoudakis et al., 2006).

### 2.19 Statistical analysis

Statistical analyses were performed using two-way analysis of variance (ANOVA) with GraphPad Prism software package (version 9.0.2). Post hoc comparisons were performed using Tukey’s multiple comparisons test. Statistical significance was set at *p < 0.05, **p < 0.01, ***p < 0.001, and ****p < 0.0001.

## 3 Results and discussion

### 3.1 DESs exhibit potent activity against antibiotic-resistant bacteria

To evaluate whether DESs exhibit antibacterial activity against antibiotic-resistant bacteria, we first determined the MIC values to assess the relative potency of each formulation. This initial assay was designed to establish which DESs were active enough to warrant further investigation and potential practical application.

The antibacterial properties of DESs were evaluated against a panel of Gram-negative bacteria harboring colistin-resistance genes, including *mcr-1* and *mcr-9*. T-glycoline and oxaline (organic acid-based DESs) exhibited notably greater antibacterial activity than non-organic acid DESs against these Gram-negative strains, with MIC values ranging from 0.42% to 0.83% (v/v). These results align with a previous report on the superior efficacy of organic acid-based DESs (Swebocki et al., 2024). T-glycoline demonstrated more potent bactericidal activity against Gram-negative bacteria compared to oxaline, as evidenced by its lower minimum bactericidal concentration (MBC) values (Table 4).

**Table 4:** MIC, MBC and MBC/MIC ratio of DESs compared to colistin against Gram-negative strains carrying *mcr* genes (*n* = 3).

Strain	Important resistance gene	MIC or MBC	T-glycoline (% v/v)	Glyceline (% v/v)	Reline (% v/v)	Oxaline (% v/v)	Colistin **(**mg/L)
*E. coli* H45	*mcr-1*	MIC	0.42	> 6.66	> 6.66	0.42	8
		MBC	0.83	> 6.66	> 6.66	> 6.66	8
		MBC/MIC ratio	1.98(+)	NA	NA	NA	1(+)
*E. coli* H66	*mcr-1*	MIC	0.42	> 6.66	> 6.66	0.42	8
		MBC	0.83	> 6.66	> 6.66	> 6.66	16
		MBC/MIC ratio	1.98(+)	NA	NA	NA	2(+)
*E. coli* H184	*mcr-1*	MIC	0.42	> 6.66	> 6.66	0.83	16
		MBC	0.42	> 6.66	> 6.66	> 6.66	16
		MBC/MIC ratio	1(+)	NA	NA	NA	1(+)
*Enterobacter cloacae* H51	*mcr-9*	MIC	0.42	> 6.66	6.66	0.21	32
		MBC	0.42	> 6.66	> 6.66	6.66	32
		MBC/MIC ratio	1(+)	NA	NA	NA	1(+)
*Salmonella enterica* H10	*mcr-1*	MIC	0.83	> 6.66	> 6.66	0.83	> 128
		MBC	0.83	> 6.66	> 6.66	> 6.66	> 128
		MBC/MIC ratio	1(+)	NA	NA	NA	NA
*Klebsiella* *variicola* H77	*mcr-9*	MIC	0.83	> 6.66	6.66	0.42	64
		MBC	0.83	> 6.66	> 6.66	> 6.66	128
		MBC/MIC ratio	1(+)	NA	NA	NA	2(+)

MBC/MIC ratio, (+) bactericidal; (−) bacteriostatic; NA - not applicable

Although colistin was found to be more effective than the tested DESs (with MICs of T-glycoline in the approximate range of 4788–9462 mg/L and of oxaline in the range of 4746–9379 mg/L), it is important to note that colistin-resistant bacteria have already emerged (Ahn et al., 2025). In contrast, no reports of resistance to DESs have been found, suggesting that they could be a promising alternative to colistin for treating drug-resistant Gram-negative pathogens.

This advantage stems from DESs have multi-target mechanisms, which include membrane disruption, protein denaturation, and induction of osmotic stress in susceptible bacterial cells (Brzeziński et al., 2025). Based on the current literature, this is the first report to demonstrate the efficacy of organic acid-based DESs against *Enterobacterales* that carry colistin resistance genes. These findings open up new possibilities for the use of DESs in medicine and food safety.

To test whether DESs can enhance the activity of colistin, we conducted checkerboard assays to evaluate the potential for synergy between T-glycoline and colistin, as well as between oxaline and colistin. This assay allowed to assess whether combining DESs with colistin improves antibacterial efficacy and reduces the required antibiotic amount.

As shown in Table 5, the fractional inhibitory concentration index (FICI) values for both combinations indicated indifference. This may be because they target different areas: colistin disrupts the outer membrane by binding to lipopolysaccharide (Sabnis et al., 2021), whereas our findings suggest that T-glycoline and oxaline may primarily affect cytoplasmic content integrity. These potential mechanistic differences might limit the synergistic potential of these combinations.

**Table 5:** Checkerboard assay for combining DESs with colistin against Gram-negative strains carrying *mcr* genes (*n* = 3).

Strains	FIC of T-glycoline in combination with colistin	FIC of oxaline in combination with colistin	FIC of colistin in combination with T-glycoline	FIC of colistin in combination with oxaline
*E. coli* H45	0.99	0.99	1.00 FICI = 1.99^a^	1.00 FICI = 1.99 a
*E. coli* H66	0.99	0.99	1.00 FICI = 1.99 a	1.00 FICI = 1.99 a
*E. coli* H184	1.98	0.99	2.00 FICI = 3.98 a	1.00 FICI = 1.99 a
*Enterobacter cloacae* H51	0.99	1.92	1.00 FICI = 1.99 a	2.00 FICI = 3.92 a
*K. variicola* H77	1.00	0.99	1.00 FICI = 2.00 a	1.00 FICI = 1.99 a

a : indifferences.

DESs containing organic acids exhibited greater antibacterial activity than conventional DESs against Gram-positive strains, with MIC values ranging from 0.21% to 0.42% (v/v) (Table 6). Oxaline displayed stronger activity than T-glycoline, as shown by its lower MIC of 0.21% (v/v). These results are consistent with previous reports showing that organic acid-based DESs are effective against all investigated strains (Swebocki et al., 2024).

**Table 6:** MIC, MBC, and MBC/MIC ratio of DESs against drug-resistant Gram-positive strains (*n* = 3).

Strain	Important resistance gene	MIC or MBC	T-glycoline (% v/v)	Glyceline (% v/v)	Reline (% v/v)	Oxaline (% v/v)
*S. aureus* USA300	Multiple	MIC	0.42	> 6.66	> 6.66	0.21
		MBC	0.42	> 6.66	> 6.66	> 6.66
		MBC/MIC ratio	1(+)	NA	NA	NA
*S. aureus* S1	Methicillin	MIC	0.42	> 6.66	> 6.66	0.21
		MBC	0.83	> 6.66	> 6.66	> 6.66
		MBC/MIC ratio	1.98(+)	NA	NA	NA
*L. monocytogenes* CIP 105,459		MIC	0.42	> 6.66	> 6.66	0.21
		MBC	0.42	> 6.66	> 6.66	> 6.66
		MBC/MIC ratio	1(+)	NA	NA	NA

MBC/MIC ratio, (+) bactericidal; (−) bacteriostatic; NA - not applicable.

The antibacterial activity of reline was evaluated against a range of Gram-positive and Gram-negative bacteria, with MIC values ranging from 6.25% to 25% (v/v) (Kocot et al., 2025), in agreement with our findings. This confirms that conventional DESs display lower antibacterial activity than organic acid-based DESs.

Furthermore, checkerboard assays were conducted to investigate the interaction between T-glycoline and oxaline. These revealed synergy against *E. coli* H184 and *Salmonella enterica* H10 (FICI = 0.49; synergy is defined as an FICI of ≤0.5). In contrast, no synergy was observed against Gram-positive bacteria (Table 7). The synergistic activity of T-glycoline and oxaline towards Gram-negative bacteria is consistent with a mechanism whereby organic acid-based DESs may acidify the periplasm and disrupt membrane integrity. This potential dual action likely results in a stronger killing effect than either component alone (Wesseling and Martin, 2022). However, further investigation is required to fully elucidate these underlying molecular pathways.

**Table 7:** Checkerboard assay for the combination of T-glycoline with oxaline against Gram-positive and Gram-negative strains (*n* = 3).

Strains	FIC T-glycoline in combination with oxaline	FIC oxaline in combination with T-glycoline	FICI
*E. coli* H184	0.247	0.278	0.496^a^
*Salmonella enterica* H10	0.248	0.248	0.496^a^
*Enterobacter cloacae* H51	0.495	0.480	0.976^b^
*K. variicola* H77	0.496	0.495	0.992^b^
*E. coli* H45	0.495	0.495	0.991^b^
*E. coli* H66	0.495	0.945	0.991^b^
*S. aureus* USA300	0.495	0.495	0.991^b^

a : synergy and ^b^: no synergy.

### 3.2 Physicochemical characterization of alginate and starch nanoparticles (NPs)

**Fig. 1:**
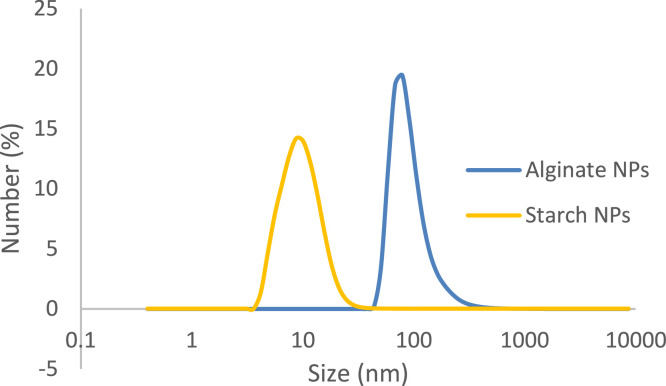
Size distribution data obtained by DLS for alginate and starch NPs.

Physicochemical characterization of NPs was performed to evaluate key properties that determine their suitability as carriers for DES delivery. Starch and alginate NPs were produced using a top-down approach involving a planetary ball mill. Their size and surface charge were then characterized using dynamic light scattering (DLS) (Zgheib et al., 2021). The size of alginate NPs was 96 nm, while that of the starch NPs was 10 nm, as shown in Fig. 1.

NPs size depends on the synthesis method. Our values are comparable to previous reports and stand out for low cost and non-toxic approach (Acharya et al., 2016). These sizes <100 nm are within the approved range of nanomaterials for medical application by the FDA. Small NP sizes- are associated with better cellular uptake and crossing conventional biological barriers (Bobo et al., 2016).

### 3.3 Nanoparticles enhance the antibacterial activity of DESs against gram-positive bacteria

We have previously demonstrated that the combination of alginate nanoparticles with small molecules and antimicrobial peptides allows to reduce the MIC values (Belguesmia et al., 2020; Hazime et al., 2022a, 2022b). In addition, recent reports evidenced that DES formulation with nanoparticles allows to enhance the stability and efficacy of water-insoluble drugs (Gonçalves et al., 2023; Sallustio et al., 2024; Zhong et al., 2024). Hence, we investigated the effect of incorporating DESs containing organic acids into alginate and starch NPs on their antibacterial activity.

Enhanced antimicrobial efficacy against Gram-positive bacteria was demonstrated by reduced minimum bactericidal concentrations (MBC) for *S. aureus* S1 when T-glycoline was combined with alginate and starch NPs. Furthermore, the MIC value for *S. aureus* USA300 decreased when oxaline was combined with alginate and starch NPs (Table 8). This suggests an interaction between the DESs and the NPs. DESs can adsorb on starch and alginate nanoparticles through polar interaction or hydrogen bonding. However, it is hard to provide the exact nature of this interaction in absence of additional characterization.

**Table 8:** Antibacterial Activity of DESs combined with alginate and starch NPs*.

Strain	MIC or MBC	T-glycoline	T-glycoline + Alginate NPs	T-glycoline + Starch NPs	Oxaline	Oxaline + Alginate NPs	Oxaline + Starch NPs
*S. aureus* S1	MIC	0.42	0.42	0.42	0.21	0.21	0.21
	MBC	0.83	0.42	0.42	> 6.66	> 6.66	> 6.66
*S. aureus* USA300	MIC	0.42	0.42	0.42	0.21	0.11	0.1
	MBC	0.42	0.83	0.83	> 6.66	> 6.66	> 6.66
*E. coli* H45	MIC	0.42	0.83	0.83	0.42	0.42	0.42
	MBC	0.83	0.83	0.83	> 6.66	> 6.66	> 6.66
*Enterobacter cloacae* H51	MIC	0.42	0.83	0.83	0.21	0.42	0.42
	MBC	0.42	0.83	0.83	6.66	> 6.66	> 6.66

⁎ Results are expressed as percentage by volume (% v/v).

In contrast, there was no significant effect on Gram-negative bacteria. This variation may be due to differences in cell wall composition. Specifically, the outer membrane of Gram-negative bacteria acts as a barrier, limiting the penetration of these compounds (Vorobyova, 2023).

### 3.4 DESs downregulate mcr-1 expression

Given the importance of colistin-resistance genes in limiting control options, the expression of *mcr*-1 in *E. coli* H45 and *mcr*-9 in *Enterobacter cloacae* H51 was studied by reverse transcriptase quantitative PCR (RT-qPCR) to evaluate the impact of DESs. This may help understand how DESs influence resistance.

**Fig. 2:**
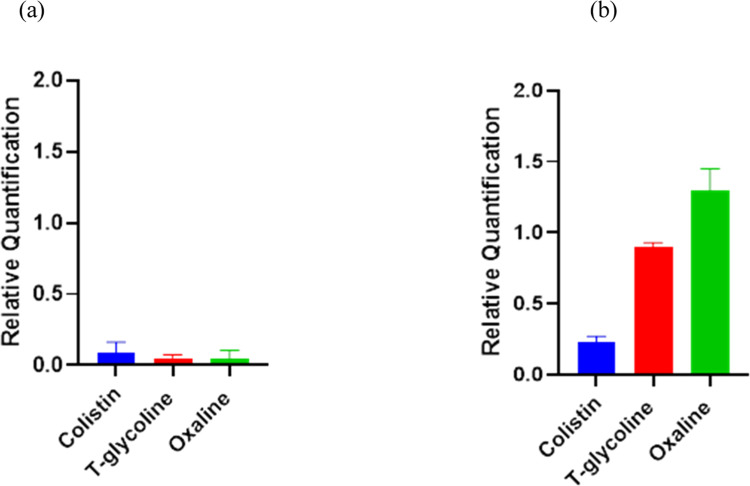
Relative expression of *mcr-1* in (a) *E. coli* H45 and (b) *mcr-9* in *Enterobacter cloacae* H51, following bacterial treatment with colistin and DESs at sub-inhibitory concentrations (MIC/2). Relative quantification (RQ) was calculated using the ΔΔCT method and normalized relative to 16S rDNA expression. Results are expressed as mean ± standard deviation. RQ greater than 2 indicates upregulation, <0.5 indicates downregulation and between 0.5 and 2 indicates no change in gene expression.

Exposure of these strains to sub-inhibitory concentrations of colistin, T-glycoline and oxaline resulted in downregulation of *mcr*-1 expression, which counters the global spread of colistin resistance mediated by *mcr*-1 on plasmids. This threatens last-resort therapy for multidrug-resistant infections. However, T-glycoline and oxaline showed no effect on *mcr*-9 expression (Fig. 2). To date, this appears to be the first study investigating the effects of DESs on the expression of antibiotic resistance genes. These findings enable novel DES-based strategies for treating and controlling resistance.

### 3.5 DESs are potent biofilm eradicators against both gram-positive and gram-negative bacteria

As biofilms on medical devices, in veterinary settings and on food preparation surfaces are clinically significant, we investigated the effectiveness of DESs in eradicating pre-established *E. coli* H45 and *S. aureus* S1 biofilms, which were selected as representative Gram-negative and Gram-positive pathogens, respectively.

**Fig. 3:**
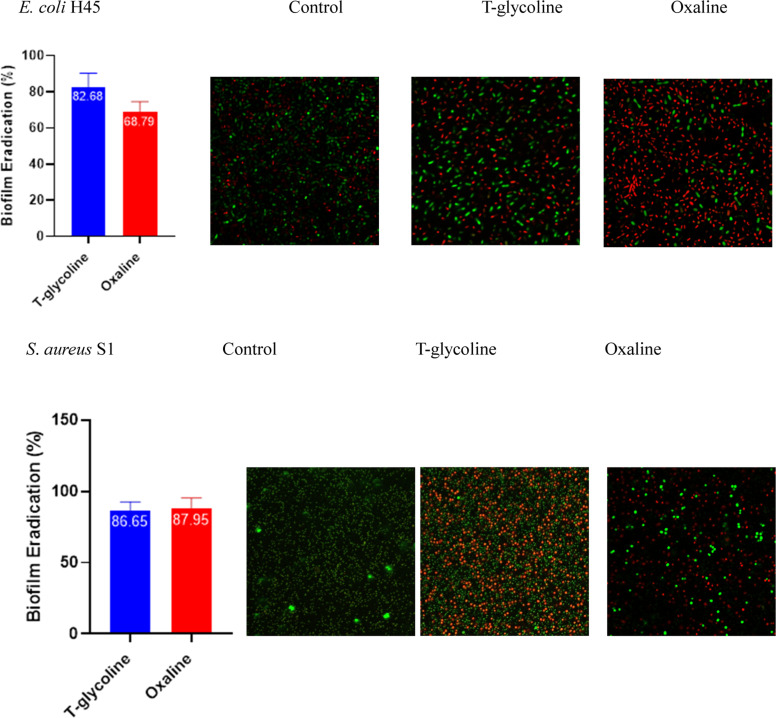
Biofilm eradication and viability in *E. coli* H45 and *S. aureus* S1 treated with DESs at 2 × MIC for 3 h. Biofilm eradication by DESs was assessed via biomass reduction quantified by crystal violet assay. Confocal live/dead staining shows Syto-9 (green, live cells) and propidium iodide (red, dead cells) in untreated controls versus DES-treated samples. The biofilms were stained directly in the Lab-Tek chambers after gentle washing and without concentration of cells prior to imaging.

Both T-glycoline and oxaline effectively eradicated established biofilms of these strains, with eradication percentages ranging from 69% to 88%. Confocal laser scanning microscopy revealed a higher proportion of dead cells in *S. aureus* S1 and *E. coli* H45 biofilms following oxaline treatment, indicating superior penetration and disruption compared to T-glycoline (Fig. 3).

In this context, neutral natural DESs, including choline chloride: xylitol and choline chloride: glycerol, significantly reduced the number of viable cells in pre-formed *S. aureus* and *P. aeruginosa* biofilms, with an average of 27–67% of *S. aureus* biofilms and 34–49% of *P*. aeruginosa biofilms being removed (Nystedt et al., 2023). These findings align with our observed potency of DESs across Gram-positive and Gram-negative strains, supporting their broad applicability in biofilm control.

### 3.6 Mode of action of DES

A time-killing assay was performed to monitor the rate of bacterial population decline upon DES exposure, in order to gain insight into the mode of action of the tested DESs. This helps distinguish between bactericidal and bacteriostatic effects, providing further evidence for the proposed mechanism.

**Fig. 4:**
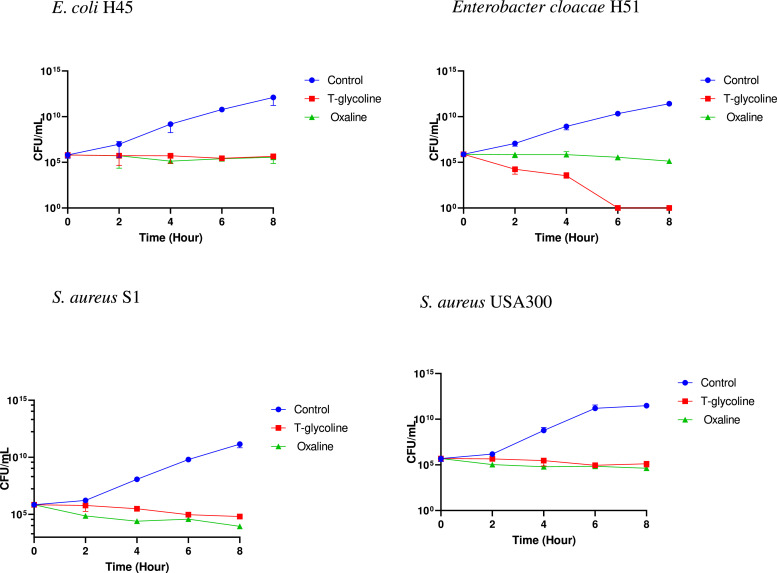
Time-killing curves for a panel of Gram-negative and Gram-positive bacteria carrying antibiotic resistance genes, following treatment with DESs at 1 × MIC.

The untreated bacterial cells of *E. coli* H45, *Enterobacter cloacae* H51, *S. aureus* S1, and *S. aureus* USA300 exhibited normal growth patterns. The DES-treated *E. coli* H45, *S. aureus* S1, and *S. aureus* USA300 maintained a constant bacterial count of approximately 10^5^ CFU/mL after 2 h of incubation, indicating a bacteriostatic action. Interestingly, *Enterobacter cloacae* H51 cells treated with T-glycoline showed a rapid decline in bacterial count, reaching approximately 0 CFU/mL after 6 h of incubation, suggesting a bactericidal action. However, treatment of *Enterobacter cloacae* H51 with oxaline resulted in a stable bacterial count of around 10^5^ CFU/mL, confirming a bacteriostatic effect (Fig. 4).

These findings are consistent with the report showing that T-glycoline and oxaline exhibit rapid antimicrobial action within 1 hour against Gram-negative bacteria, but require a longer exposure time and higher concentrations to achieve >99.9% suppression of Gram-positive strains (Swebocki et al., 2024). This explains the observed bactericidal effect of T-glycoline specifically against the Gram-negative *Enterobacter cloacae* H51 strain. Furthermore, the bactericidal action of T-glycoline against *Enterobacter cloacae* H51 is probably due to differences in outer membrane permeability and fluidity (Kim et al., 2020).

**Fig. 5:**
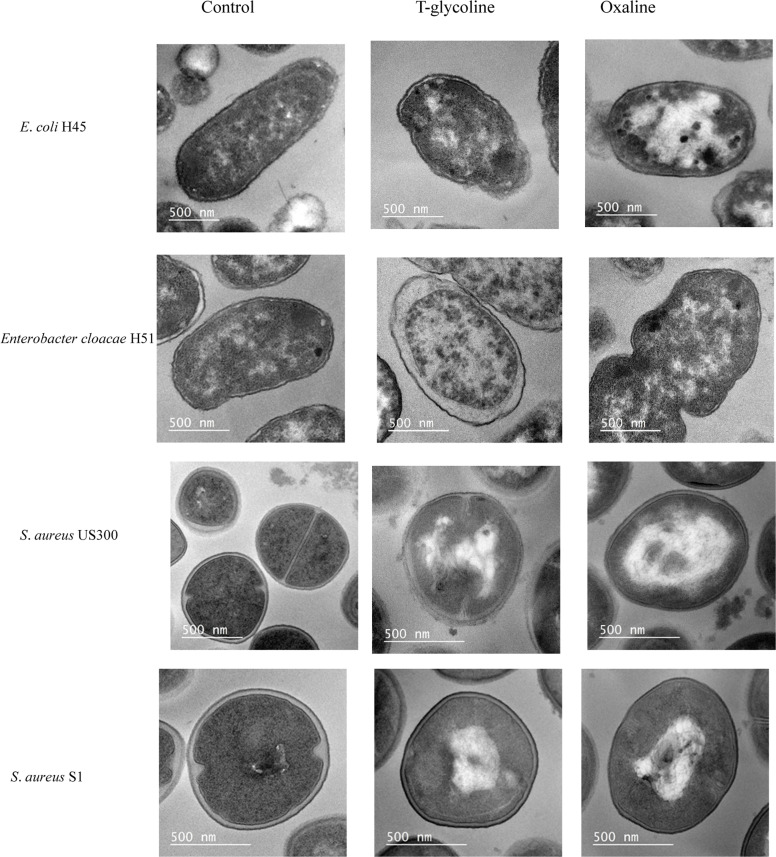
TEM observation of Gram-negative and Gram-positive bacteria. Comparison between untreated control cells and cells treated with T-glycoline and oxaline at 1 × MIC.

Transmission electron microscopy (TEM) analysis was used to visualize the structural impact of DESs on bacterial cells, providing morphological evidence that supports the proposed mode of action. TEM analysis revealed that untreated bacterial cells maintained their characteristic shape and uniformity, with well-preserved morphology, intact cell walls, and uniform cytoplasmic contents. In contrast, DES-treated cells exhibited pronounced ultrastructural alterations in the tested bacterial cells. These alterations indicated significant cellular stress and cytoplasmic integrity disruption. Furthermore, extensive loss of intracellular content was observed in *Enterobacter cloacae* H51 treated with T-glycoline, which corroborates the time-kill assay results and confirms that T-glycoline exerts a bactericidal mode of action on *Enterobacter cloacae* H51 (Fig. 5). Bacterial cell wall disruption following treatment with organic DESs at 8 × MIC concentrations has been documented (Swebocki et al., 2024).

**Fig. 6:**
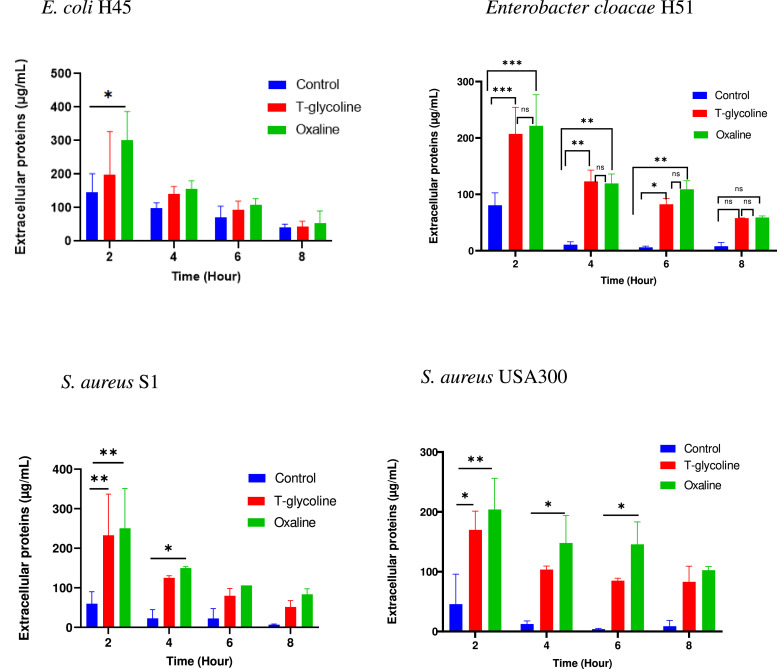
Intracellular protein release from a panel of Gram-negative and Gram-positive antibiotic-resistant bacteria following DES treatment at 1 × MIC. Data represent mean values from triplicate experiments.

To better understand the mode of action of DESs, we quantified the amount of protein released after treatment of Gram-negative bacteria (*E. coli* H45 and *Enterobacter cloacae* H51) and Gram-positive bacteria (*S. aureus* S1 and *S. aureus* USA300), as a measure of membrane damage. Protein release was minimal relative to the control in most strains, except for *Enterobacter cloacae* H51, which recorded a significant increase in protein release starting at 2 h (Fig. 6). These findings indicate that DESs cause limited membrane disruption across most strains, with a notably stronger effect observed in *Enterobacter cloacae* H51. This observation is consistent with a previous study that has reported cell wall disruption following the treatment of bacterial cells with organic DESs (Swebocki et al., 2024).

**Fig. 7:**
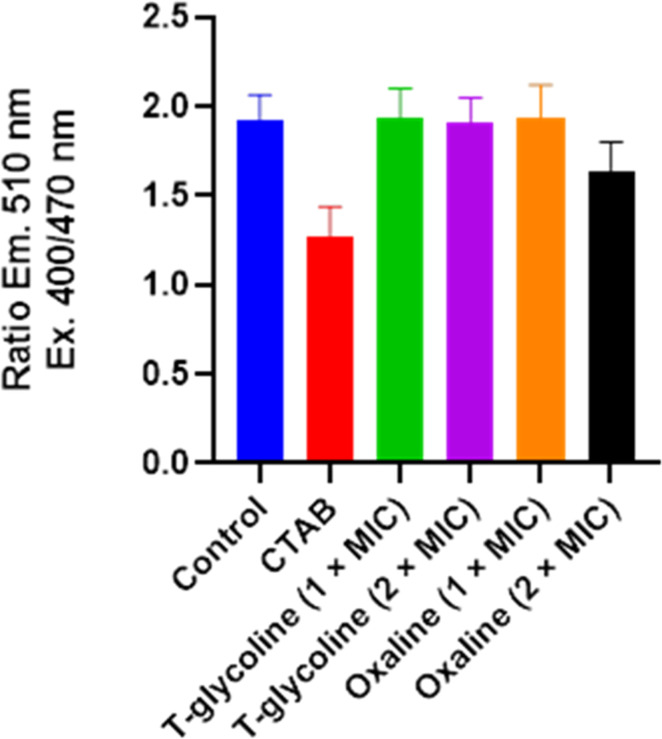
Assessment of the membrane-damaging activity of DESs using the ratiometric fluorescence response of *L. monocytogenes* EGD-e/pNZ-Phelp-pHluorin. CTAB (0.01%) was used as a positive control, while untreated bacteria cultured in *Listeria* minimal buffer (LMB) served as the negative control. Data represent the mean values from triplicate experiments, with error bars indicating standard deviation.

To study the ability of DESs to induce pore formation, we monitored the response of a ratiometric pH-sensitive variant of green fluorescent protein (GFP), expressed in *L. monocytogenes* EGD-e/pNZ-Phelp-pHluorin, to cetyltrimethylammonium bromide (CTAB), a well-known pore-forming detergent, which resulted in a decrease in the fluorescence excitation ratio (400/470 nm). In contrast, treatment with DESs at 1 × and 2 × MIC did not induce any significant change in the fluorescence ratio, indicating an absence of pore formation in *L. monocytogenes*. These results are consistent with those of the time-kill assay, TEM observations, and protein release quantification. Collectively, they confirm that DESs do not exhibit pore-forming activity or disrupt the cell wall integrity of Gram-positive bacteria (Fig. 7).

### 3.7 DESs exhibit virucidal activity against enveloped and non-enveloped virus

**Fig. 8:**
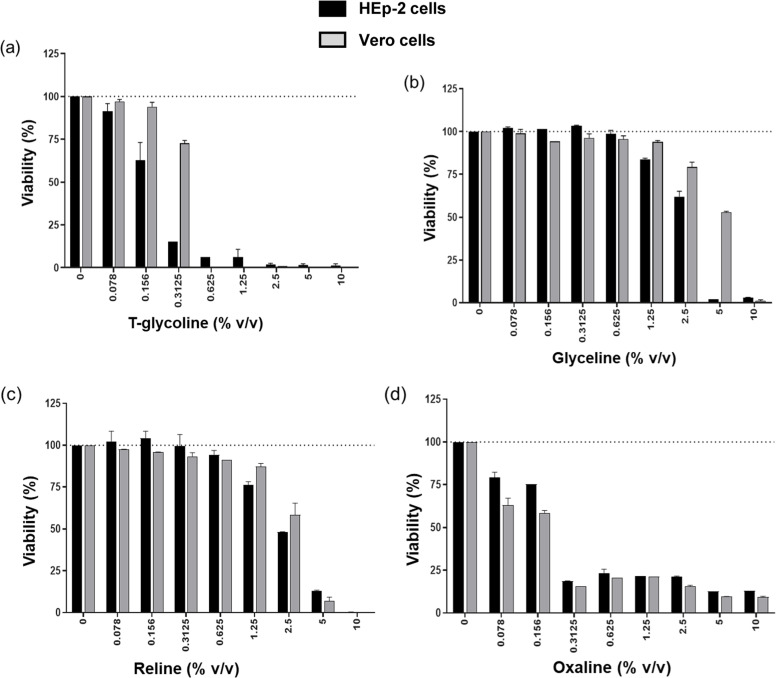
Cytotoxicity of DESs on HEp-2 and Vero cells. Various dilutions of (a) T-glycoline, (b) glyceline, (c) reline and (d) oxaline diluted (% v/v) in MEM were added to 2.5 × 10^4^ HEp-2 or Vero cells to determine cytotoxic concentrations. After 24 h of incubation, cell viability was assessed using the Orangu™ assay and expressed as a percentage compared with untreated cells. The results are presented as mean ± SD of two independent experiments.

To examine the ability of DESs to act beyond bacteria, we tested their activity against both enveloped and non-enveloped viruses known to be responsible for healthcare-associated infections. The cytotoxic effect of DESs on HEp-2 and Vero cells was evaluated by measuring cell viability after 24 h of incubation. All DESs at 10% (v/v) completely reduced the viability of the above-mentioned cell lines. T-glycoline and oxaline exhibited higher cytotoxicity towards HEp-2 and Vero cells compared to glyceline and reline at the same concentrations. The 50% cytotoxic concentration (CC_50_) values obtained with T-glycoline were 0.16 ± 0.07% and 0.33 ± 0.11% in HEp-2 and Vero cell cultures, respectively. The CC50 values recorded with glyceline were 2.64 ± 1.06% and 4.84 ± 2.27% in HEp-2 and Vero cell cultures, respectively. The CC50 values acquired with reline were 2.32 ± 1.18% and 2.49 ± 1.15% in HEp-2 and Vero cell cultures, respectively. The CC50 values obtained with oxaline were 0.22 ± 0.11% and 0.14 ± 0.08% in HEp-2 and Vero cell cultures, respectively (Fig. 8).

**Fig. 9:**
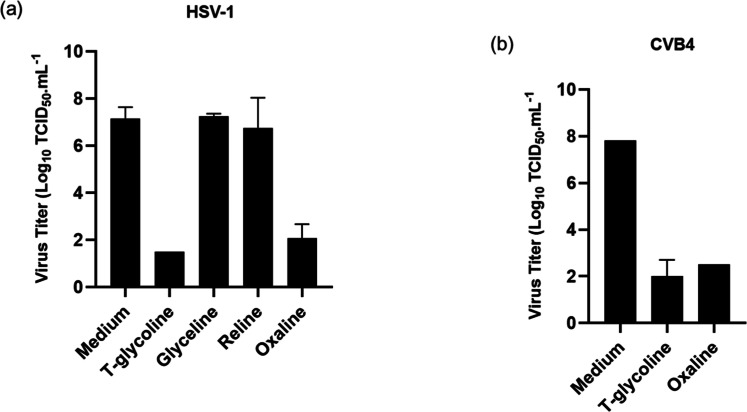
Levels of infectious particles after exposure to DESs. DESs at 10% (v/v) were incubated with HSV-1 (a) or CVB4 (b) for one hour. The mixtures were then diluted 1:10 to overcome the cytotoxic concentration before virus titration on HEp-2 or Vero cell cultures using the end-point dilution assay. The viral titer is expressed as TCID_50_/mL. The results are presented as mean ± SD of two independent experiments.

The virucidal activity of DESs at 10% was first evaluated against HSV-1, an enveloped virus, following 1 hour of exposure. The cytotoxic effect of the mixture DES/HSV-1 on cells was then reduced by dilution (1/10) before virus titration to avoid residual cytotoxicity artifact. The viral titers were not obviously different when HSV-1 was incubated in the presence of glyceline or reline (7.2 ± 0.1 and 6.7 ± 1.2 log_10_ TCID_50_/mL) as compared to that of the untreated virus (7.6 ± 0.4 log_10_ TCID_50_/mL). In contrast, a strong reduction in infectious titers of 5.7 and 5.1 log_10_ TCID_50_/mL was obtained, when the virus was incubated in the presence of T-glycoline and oxaline, respectively (Fig. 9a). Thereafter, the virucidal activity of T-glycoline and oxaline against coxsackievirus B4 E2 strain, a non-enveloped virus was evaluated according to the same protocol. Interestingly, T-glycoline and oxaline reduced the viral titer of CVB4 by 5.8 and 5.3 log_10_ TCID_50_/mL, respectively (Fig. 9b).

Our observations on the virucidal activity of DESs, particularly T-glycoline and oxaline, against HSV-1 are consistent with previous reports of significant reductions in viral infectivity of enveloped viruses such as HSV-1, HSV-2 (Zakrewsky et al., 2016), and human coronavirus hCoV229E (Shevachman et al., 2022) after exposure to DESs. It has been suggested that DESs have a virucidal effect via disruption of the viral envelope (Shevachman et al., 2022). Interestingly, to our knowledge, this is the first study reporting a virucidal effect of DESs on CVB4, a non-enveloped virus. The DESs tested in this study are mixtures of quaternary ammonium salts and other products. The virucidal mode of action of T-glycoline and oxaline towards CVB4, a non-enveloped virus, deserves further investigation since it is known that quaternary ammonium salts by themselves exhibit no virucidal activity against such viruses (Tuladhar et al., 2012). Moreover, the impact of short time exposure to DESs on viral infectivity deserves further study.

### 3.8 Safety consideration

The attractive features of DESs make them highly suitable for a variety of applications, including those in the health and food industries. However, multiple studies have emphasised the importance of thoroughly evaluating their toxicological profiles (Macário et al., 2019).

**Fig. 10:**
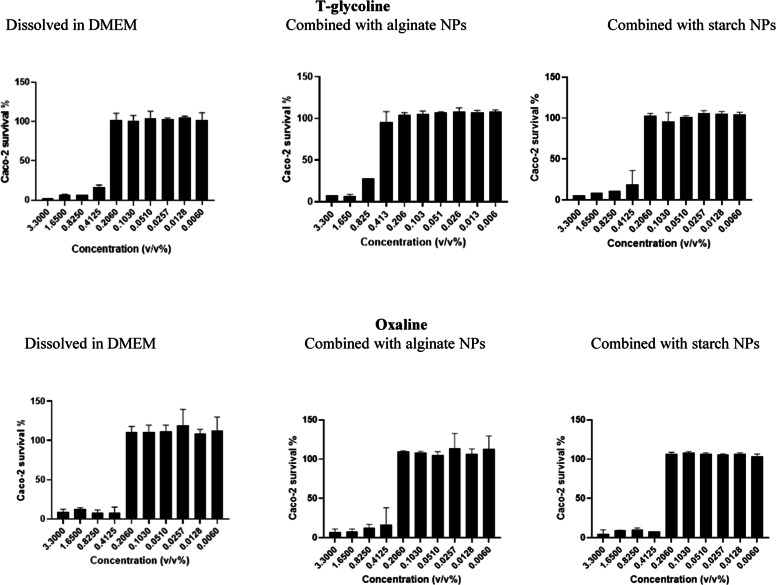
Effects of DESs on Caco-2 cell viability. Cells were incubated for 24 h with DES-incorporated alginate, starch, or yeast nanoparticles dispersed in DMEM at decreasing concentrations (0.006–3.3%, v/v). Cell viability was assessed by measuring mitochondrial dehydrogenase activity using the CCK-8 assay. Data are presented as mean ± SD (N = 3, n = 3).

To evaluate the cytotoxic effects of DESs on Caco-2 cells, T-glycoline was tested at its MIC (0.42%) value. At this concentration, T-glycoline resulted in very low cell viability when dissolved in Dulbecco's Modified Eagle's Medium (DMEM) or when incorporated into starch NPs. In contrast, incorporation of T-glycoline into alginate NPs at the same MIC restored cell viability to approximately 95 ± 8.95%. The MIC of oxaline against Gram-positive bacteria, including *S. aureus*, was 0.21%, at which cell viability remained close to 100%, regardless of whether oxaline was dissolved in DMEM or incorporated into NPs. However, the MIC of oxaline against Gram-negative bacteria was higher (0.42%), and at this concentration, cell viability was markedly reduced for oxaline in DMEM as well as across all NP formulations (Fig. 10). Furthermore, the cytotoxic effect of DESs on HEp-2 and Vero cells was evaluated as described in Section 3.7.

The cytotoxicity of DESs has previously evaluated in various cell lines, including L929 fibroblast cells (Paiva et al., 2014), A375 human malignant melanoma (Hayyan et al., 2015), MCF-7 human breast cancer (Mbous et al., 2017), and CCO fish cells (Radošević et al., 2015). Consistent with our findings, choline chloride-based DESs have been reported to exhibit relatively high cytotoxicity across multiple cell models (Hayyan et al., 2015). Furthermore, specific DES formulations, such as choline chloride: oxalic acid, have been described as moderately toxic (Radošević et al., 2015).

Notably, T-glycoline combination with alginate NPs markedly reduced DES-induced toxicity, highlighting the protective role of polymeric carriers (Hoseinzadeh et al., 2023; Pedro et al., 2022). In contrast, alginate NPs did not alleviate the cytotoxicity associated with oxalic acid-based DESs, likely due to acid-driven mechanisms that are not effectively mitigated by such carriers (Macário et al., 2019)

**Fig. 11:**
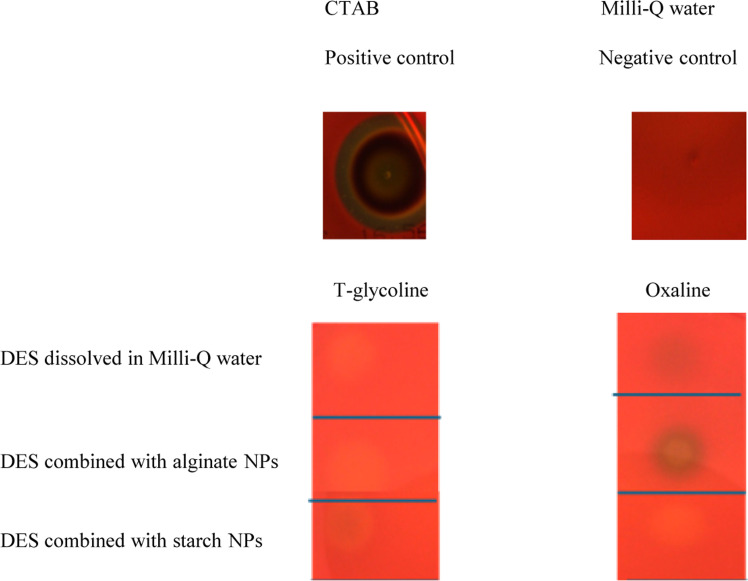
Evaluation of the haemolytic activity of DESs on sheep blood. The DESs were dissolved in Milli-Q water and incorporated into alginate and starch NPs. CTAB served as the positive control, while Milli-Q water was used as the negative control.

To further evaluate the toxicity of our DES formulations, haemolytic activity was assessed using sheep blood agar, with CTAB serving as the positive control and Milli-Q water as the negative control. Neither T-glycoline nor oxaline exhibited haemolytic effects when dissolved in Milli-Q water or incorporated into NPs. However, the incorporation of oxaline with alginate NPs caused slight brown discolouration of the blood agar, indicative of minor oxidative changes consistent with α-haemolysis-like methaemoglobin formation (Fig. 11).

While our findings highlight the potential of T-glycoline and oxaline as antimicrobial agents, achieving a balance between high efficacy and minimal mammalian cytotoxicity remains challenging. Our results show that these formulations exhibit significant cytotoxicity toward Caco-2 cells at concentrations effective against targeted pathogens. Although the integration of alginate-based nanoparticles improved the safety profile, simultaneous optimization of antimicrobial potency and cellular compatibility has not yet been fully achieved. Future work will focus on refining these formulations to increase the safety margin and ensure that these DES-based systems can be used safely in practical applications.

## 4 Conclusion

The presence of antibiotic-resistant bacteria, biofilms, and viral contamination on medical devices and in food processing poses a serious threat to infection control. Our study shows that deep eutectic solvents (DESs), T-glycoline and oxaline formed using organic acids, exhibit strong antimicrobial activity against antibiotic-resistant bacteria, such as methicillin-resistant *S. aureus* (MRSA), as well as colistin-resistant *Enterobacteriaceae*, and against viruses, including the non-enveloped coxsackievirus B4. Furthermore, alginate-based nanoparticles exhibited strain-dependent effects, enhancing the activity of T-glycoline against Gram-positive bacteria and improving its safety profile. It should be noted that the range of bacterial and viral strains tested, as well as the in vitro conditions used, do not fully reflect the complexity of practical application. Future molecular studies should explore in more detail how T-glycoline and oxaline affect bacterial cells. Additionally, the interaction mechanisms between nanoparticles and DESs should be investigated. Finally, advanced encapsulation strategies should be investigated to reduce cytotoxicity and improve formulation stability. This would facilitate the effective development of DES formulations for industrial use.

## Funding

The authors acknowledge the support of the Hauts-de-France region in funding Dr. Mohamed Maky's postdoctoral position within the CPER-FEDER BiHauts Eco de France programme. The authors would also like to thank the Ministry of National Education, Research and Technology, the 10.13039/100012868University of Lille and the Lille University Hospital. Chérifa Obone Opimba is supported by Agence Nationale des Bourses du Gabon scholarship No. 1800603.

## Declaration of competing interest

The authors declare the following financial interests/personal relationships which may be considered as potential competing interests: Djamel Drider reports financial support was provided by Hauts-de-France Region. Djamel Drider reports a relationship with Hauts-de-France Region that includes: funding grants. No patent, no thing to declare If there are other authors, they declare that they have no known competing financial interests or personal relationships that could have appeared to influence the work reported in this paper.

## Data Availability

The data that support the findings of this study are available from the corresponding author upon request.
